# Reviewers in 2022

**DOI:** 10.1098/rspb.2023.0382

**Published:** 2023-03-29

**Authors:** Proceedings B Editorial Team

The Editors of Proceedings B would like to thank all reviewers who contributed their time by refereeing manuscripts submitted throughout 2022. From previous feedback, we know that most reviewers value recognition of their efforts by the journal and through services, such as Web of Science Reviewer Recognition Service (previously Publons), which is integrated with Proceedings B.

To provide an opportunity for recognition, we list the names of all reviewers (unless they have opted out). This article is made permanent and citeable by its digital object identifier (DOI). We encourage you to quote your contribution in your applications for tenure and grants or other forms of research assessment. Where you have provided it, we have included your ORCID, so your contribution can be unambiguously assigned to you.

The team really does appreciate all the work our reviewers do on behalf of the journal, and we look forward to working with you again in 2023.

Aanen D https://orcid.org/0000-0002-5702-1617

Aartsma Y

Abbott KC

Abe J

Abouheif E https://orcid.org/0000-0001-7651-1737

Abreu C

Accatino https://orcid.org/0000-0002-6719-8539

Accatino F

Ackerley R https://orcid.org/0000-0003-4621-7929

Ackland G https://orcid.org/0000-0002-1205-7675

Adamala K

Adler F

Agneesh B https://orcid.org/0000-0002-8347-2171

Agostina T

Aguilera F https://orcid.org/0000-0003-3235-931X

Aidan W https://orcid.org/0000-0001-5997-8001

Ainley DG

Aizen M

Akcay C https://orcid.org/0000-0003-0635-9586

Akle V https://orcid.org/0000-0002-2050-5511

Alaasam V https://orcid.org/0000-0003-1670-6780

Albajes R

Albantakis L

Albery GF https://orcid.org/0000-0001-6260-2662

Albrecht J https://orcid.org/0000-0002-9708-9413

Albrecht T https://orcid.org/0000-0002-9213-0034

Alejandro L

Alexander AS https://orcid.org/0000-0002-0483-5748

Alexis P

Alison O

Alizon S https://orcid.org/0000-0002-0779-9543

Allan E

Allen J https://orcid.org/0000-0002-0950-0429

Allen K

Allen M https://orcid.org/0000-0002-6632-4337

Almeida-Santos S

Aluru N

Alyokhin A

Amanda H

Amato K

Amy B

Andersen K https://orcid.org/0000-0002-8478-3430

Andersen M

Anderson J

Anderson P https://orcid.org/0000-0001-7133-8322

Anderson S https://orcid.org/0000-0003-3096-1120

Anderson T

Andersson M

Andrade S

André J-B https://orcid.org/0000-0001-9069-447X

Andrea F

Andrews C

Angelini C

Angielczyk K

Annapaola R https://orcid.org/0000-0003-3997-6783

Ansorge R

Anthes N

Antia R

Aracely M

Aranda I

Araujo W

Arceo-Gomez G

Archie E https://orcid.org/0000-0002-1187-0998

Arikawa K https://orcid.org/0000-0002-4365-0762

Aristide L

Armitage S

Armstrong EE

Armstrong E https://orcid.org/0000-0003-1223-4907

Armstrong P

Armstrong T

Arnedo M https://orcid.org/0000-0003-1402-4727

Arnot M

Arnqvist G https://orcid.org/0000-0002-3501-3376

Arpita N

Bengisu S

Asgari S

Ashby B https://orcid.org/0000-0001-5588-7081

Ashish BG https://orcid.org/0000-0002-6923-3596

Ashton L

Athreya R

Atwater D

Aubier T https://orcid.org/0000-0001-8543-5596

Audet J-N

Audzijonyte A

Auer T

Auerbuch V

August E-C

Auld J

Austad S

Austen E

Avani M https://orcid.org/0000-0003-1443-5737

Avaria-Llautureo J

Axelrod C http://orcid.org/0000-0002-6130-0168

Aya G https://orcid.org/0000-0003-2291-2222

Ayali A https://orcid.org/0000-0001-6152-2927

Aznar FJ

Azzellino A https://orcid.org/0000-0003-1065-9469

Baalousha M

Baguette M

Bahr K

Bahram M

Baird E https://orcid.org/0000-0003-3625-3897

Bairlein F

Baker R https://orcid.org/0000-0002-2661-8103

Bakker T

Balamurali MG

Balard A https://orcid.org/0000-0002-0942-7479

Balasubramaniam R https://orcid.org/0000-0001-7575-3080

Baldan D

Baliga V https://orcid.org/0000-0002-9367-8974

Balseiro D https://orcid.org/0000-0003-3015-9066

Balthazart J https://orcid.org/0000-0001-9492-2126

Banerjee T

Banks-Leite C

Baquero F

Barbero F

Barbosa M https://orcid.org/0000-0003-0327-9580

Barbour M

Barclay P https://orcid.org/0000-0002-7905-9069

Bard K

Bardin J https://orcid.org/0000-0003-2382-4259

Barlow D https://orcid.org/0000-0001-6102-5058

Barrett B https://orcid.org/0000-0002-2725-2385

Barrett L https://orcid.org/0000-0003-1841-2997

Barrett R

Barta Z

Barth J

Barton N

Barua A https://orcid.org/0000-0002-8347-2171

Barve S https://orcid.org/0000-0001-5840-8023

Bassing S https://orcid.org/0000-0001-6295-6372

Batáry P

Bates K

Bateson M https://orcid.org/0000-0002-0861-0191

Baudier K https://orcid.org/0000-0001-8450-3788

Baumgartner A

Bayer P https://orcid.org/0000-0001-8530-3067

Bayliss A

Beavan A

Beck K https://orcid.org/0000-0002-5027-0207

Beck R https://orcid.org/0000-0002-7050-7072

Beckerman A

Beckman N

Bedhomme S

Beechler B https://orcid.org/0000-0002-9711-4340

Beetz MJ

Behrens M https://orcid.org/0000-0003-2082-8860

Beik M

Beja-Pereira A https://orcid.org/0000-0002-1607-7382

Bell A https://orcid.org/0000-0001-8933-8494

Bell G https://orcid.org/0000-0002-7280-6391

Bello Fde

Bellworthy J https://orcid.org/0000-0003-2674-2750

Beltran RS https://orcid.org/0000-0002-8520-1105

Benayas J

Ben-Horin T

Bennett E

Bennett J

Bennington C

Ben-Shahar Y

Benson-Amram S https://orcid.org/0000-0003-0147-7559

Benton M https://orcid.org/0000-0002-4323-1824

Bentz A https://orcid.org/0000-0003-2445-4886

Berbel-Filho W https://orcid.org/0000-0001-6991-4685

Berger M

Bergholz K

Bernal M

Bernauer O https://orcid.org/0000-0002-4814-3188

Berthaume M https://orcid.org/0000-0003-1298-242X

Bertram J

Bertram M https://orcid.org/0000-0001-5320-8444

Berv J

Bestion E https://orcid.org/0000-0001-5622-7907

Bethany H https://orcid.org/0000-0001-9502-5582

Bety J

Beutel R

Bhullar B-A

Bicknell R https://orcid.org/0000-0001-8541-9035

Biewener A https://orcid.org/0000-0003-3303-8737

Bilde T

Bissett P

Bjork J

Björkman C

Blake P https://orcid.org/0000-0003-3968-9880

Blakeslee A https://orcid.org/0000-0001-9667-2175

Blamires S https://orcid.org/0000-0001-5953-3723

Blanchet S https://orcid.org/0000-0002-3843-589X

Blanckenhorn W https://orcid.org/0000-0002-0713-3944

Blankers T https://orcid.org/0000-0002-1893-8537

Blount Z

Boardman L

Bodey T https://orcid.org/0000-0002-5334-9615

Boeckx C

Boersma J

Boettiger C

Boggs C

Bohn M https://orcid.org/0000-0001-6006-1348

Bohnert KA

Boipelo T-M

Bokony V

Bolenz F

Bollazzi M https://orcid.org/0000-0003-3361-5602

Bonier F https://orcid.org/0000-0001-7245-0244

Bonneaud C https://orcid.org/0000-0003-2248-3288

Bonnet T

Bonter D

Booth D https://orcid.org/0000-0002-8256-1412

Booth W https://orcid.org/0000-0003-2355-0702

Boots M https://orcid.org/0000-0003-3763-6136

Borah B

Boratynski Z https://orcid.org/0000-0003-4668-4922

Bordenstein S https://orcid.org/0000-0001-7346-0954

Bose A https://orcid.org/0000-0001-5716-0097

Botías C https://orcid.org/0000-0002-3891-9931

Bottery MJ https://orcid.org/0000-0001-5790-1756

Bötzl F

Boulestin B https://orcid.org/0000-0002-0820-795X

Bourdeau PE

Bourke A

Bourne D

Bowden T

Bowers D

Bowers E https://orcid.org/0000-0003-4488-1463

Bowman E

Bowsher J https://orcid.org/0000-0001-6347-9635

Boyle M

Bradley D

Braga M https://orcid.org/0000-0002-1253-2536

Brandley N

Bräuer J

Braulik G

Braun J https://orcid.org/0000-0003-2359-1838

Bray E https://orcid.org/0000-0002-3230-0636

Bredeche N https://orcid.org/0000-0002-8241-7461

Brennan P

Brennan R

Bretman A https://orcid.org/0000-0002-4421-3337

Brewer S

Brigitte T https://orcid.org/0000-0003-3801-3192

Brink K https://orcid.org/0000-0001-6630-1525

Brisson J https://orcid.org/0000-0001-7000-0709

Brocklehurst N https://orcid.org/0000-0003-2650-5480

Broder D

Broderick C https://orcid.org/0000-0001-5944-1706

Broman K https://orcid.org/0000-0002-4914-6671

Brommer J https://orcid.org/0000-0002-2435-2612

Broom M

Brown C

Brown C https://orcid.org/0000-0002-0210-1820

Brown W

Buckley J https://orcid.org/0000-0003-2264-4096

Budischak S

Bueti D

Buetti S

Buggs R

Burbrink F https://orcid.org/0000-0001-6687-8332

Burchill A

Burdfield-Steel E

Burdon F

Burghardt L

Burin G

Burke M

Burmeister S https://orcid.org/0000-0001-5612-692X

Burnett E

Burr D https://orcid.org/0000-0003-1541-8832

Burraco P https://orcid.org/0000-0002-9007-2643

Burrell B https://orcid.org/0000-0002-2562-6072

Burslem D

Burton T https://orcid.org/0000-0002-0215-0227

Burton-Chellew M https://orcid.org/0000-0002-5330-0379

Buschbeck E

Büscher J https://orcid.org/0000-0003-2399-4020

Bush A

Buss D

Butlin R

Butterfield N https://orcid.org/0000-0002-3046-7520

Button D

Byrne M https://orcid.org/0000-0002-8902-9808

Byrne M

Cadena E

Cai C https://orcid.org/0000-0002-9283-8323

Cailin C

Caillaud D

Calosi P

Calovi D https://orcid.org/0000-0002-2452-9801

Camacho C https://orcid.org/0000-0002-9704-5816

Camarero JJ https://orcid.org/0000-0003-2436-2922

Camerlink I https://orcid.org/0000-0002-3427-2210

Cameron SA https://orcid.org/0000-0003-3212-3294

Campbell C https://orcid.org/0000-0003-3770-7432

Campbell P https://orcid.org/0000-0001-7660-9814

Campennì M https://orcid.org/0000-0001-7649-4869

Campione N

Campobello D https://orcid.org/0000-0003-4094-9395

Campos F https://orcid.org/0000-0001-9826-751X

Campos P https://orcid.org/0000-0003-1285-4671

Camus M https://orcid.org/0000-0003-0626-6865

Candidi M

Cañestro C

Cantalapiedra J https://orcid.org/0000-0003-0913-7735

Cantarero A https://orcid.org/0000-0002-5816-701X

Cantin N

Capellini I

Capilla-Lasheras P https://orcid.org/0000-0001-6091-7089

Cardoso G https://orcid.org/0000-0001-6258-1881

Carere C

Carlson J

Carlucci M https://orcid.org/0000-0002-5868-7090

Carnegie R

Caro T https://orcid.org/0000-0001-6804-8519

Carrier D

Carroll T

Carstens B

Carter G

Cartwright P

Casacci L

Casagrande S https://orcid.org/0000-0002-4264-8062

Casas J https://orcid.org/0000-0003-1666-295X

Castiglione S

Catenazzi A

Catherine M

Cavedon M

Cavers S https://orcid.org/0000-0003-2139-9236

Caves E https://orcid.org/0000-0003-3497-5925

Cayol C

Cayuela H https://orcid.org/0000-0002-3529-0736

Cazelles B

Ceccarelli D

Centler F

Cerritelli G

Champer J https://orcid.org/0000-0002-3814-3774

Chang C-C

Chapman D

Chapuisat M https://orcid.org/0000-0001-7207-199X

Chari LD

Charpentier C

Chay H

Chen KF

Cheng K https://orcid.org/0000-0002-4913-2691

Chenoweth S https://orcid.org/0000-0002-8303-9159

Chentao W https://orcid.org/0000-0001-7310-0473

Chevin L-M https://orcid.org/0000-0003-4188-4618

Chiarenza A https://orcid.org/0000-0001-5525-6730

Chimento M https://orcid.org/0000-0001-5697-1701

Chiocchio A https://orcid.org/0000-0002-0067-7025

Chiou K

Chipman A https://orcid.org/0000-0001-6696-840X

Chippindale AK

Chomicki G

Chorus I

Chouvenc T https://orcid.org/0000-0003-3154-2489

Chow PKaY https://orcid.org/0000-0002-8208-592X

Christina W https://orcid.org/0000-0003-0069-3014

Christy J

Chung H https://orcid.org/0000-0001-5056-2755

Cillov A

Civitello D https://orcid.org/0000-0001-8394-6288

Claidiere N https://orcid.org/0000-0002-4472-6597

Claramunt S https://orcid.org/0000-0002-8926-5974

Clark N

Clark-Hachtel C

Clement C https://orcid.org/0000-0002-8421-1029

Clements CF https://orcid.org/0000-0001-5677-5401

Clements C https://orcid.org/0000-0002-0637-1625

Clifton S https://orcid.org/0000-0001-5949-068X

Clink D

Cloyed C https://orcid.org/0000-0001-6321-5808

Codd J https://orcid.org/0000-0003-0211-1786

Cody P

Coelho A https://orcid.org/0000-0002-5315-8616

Cohen E

Cohen H https://orcid.org/0000-0001-6188-3079

Cole S https://orcid.org/0000-0001-6432-8994

Collet J

Collins D

Collins S https://orcid.org/0000-0003-3856-4285

Colston J https://orcid.org/0000-0003-4567-5813

Comeron J https://orcid.org/0000-0002-1256-6437

Concepción E

Condamine F https://orcid.org/0000-0003-1673-9910

Condron B https://orcid.org/0000-0003-2610-5065

Cong P-Y https://orcid.org/0000-0001-9910-6478

Connallon T https://orcid.org/0000-0002-1962-4951

Conti-Jerpe I

Conti-Jerpe IE

Contolini G https://orcid.org/0000-0001-5839-5330

Cook J https://orcid.org/0000-0002-7320-1706

Cooke R https://orcid.org/0000-0003-0601-8888

Cooney C https://orcid.org/0000-0002-4872-9146

Cooney D https://orcid.org/0000-0001-8643-0893

Cooper B

Cooper R https://orcid.org/0000-0001-7234-4361

Corby V

Corrales-Aguilar E

Corsini M

Costanzo A

Costa-Pereira R https://orcid.org/0000-0003-2370-5866

Coulson T https://orcid.org/0000-0001-9371-9003

Courtiol A

Cousseau L https://orcid.org/0000-0001-9719-0434

Cracraft J

Crall J

Crandall E https://orcid.org/0000-0001-8580-3651

Crates R

Criscione C

Crockford C

Crompton R

Cronberg N

Cronin A https://orcid.org/0000-0003-2075-1138

Cronin T https://orcid.org/0000-0001-7375-9382

Croshaw DA

Crowe-Riddell J https://orcid.org/0000-0003-2794-2914

Crump A https://orcid.org/0000-0003-4485-5740

Cryan P

Csergo A

Cuello W https://orcid.org/0000-0002-9464-3802

Cueva del Castillo R https://orcid.org/0000-0002-6385-0729

Cunningham S https://orcid.org/0000-0003-0703-6893

Curley J https://orcid.org/0000-0001-5546-007X

Currie S https://orcid.org/0000-0001-5956-0481

Cybulski J

Czaczkes T https://orcid.org/0000-0002-1350-4975

Czarkwiani A

Dadda M

D'Alba L https://orcid.org/0000-0002-2478-3455

Dallas T https://orcid.org/0000-0003-3328-9958

Dalmaijer E https://orcid.org/0000-0003-3241-0760

Dammhahn M

Daniel M

Daniel P

daniele C

Danielle ST

Danny M

Dantzer B https://orcid.org/0000-0002-3058-265X

Darling E

Darragh K

Darroch S

Darveau C-A

Das S

Daskalova G

Davalos L

Davenport RJ https://orcid.org/0000-0002-6828-9846

Davesne D https://orcid.org/0000-0002-4775-2360

David B https://orcid.org/0000-0002-1492-8710

David W https://orcid.org/0000-0001-5163-8096

David Z

Davidson G

Davis C https://orcid.org/0000-0001-6187-9010

Davis G https://orcid.org/0000-0003-3312-4942

Davis M

Davoren G

Dawson W

De Agrò M https://orcid.org/0000-0001-9284-5964

de Anna P https://orcid.org/0000-0002-1492-5755

De Baets K https://orcid.org/0000-0002-1651-321X

de Castro E https://orcid.org/0000-0002-4731-3835

De Chiara M

De Groote F https://orcid.org/0000-0002-4255-8673

de Hevia M-D

de la Pena E

De la Rua P https://orcid.org/0000-0002-0058-1402

De Moor D https://orcid.org/0000-0003-2474-6125

Dearborn D

Death R

Debapryio C

Debarre F https://orcid.org/0000-0003-2497-833X

Debes P https://orcid.org/0000-0003-4491-9564

Debray R

DeCasien A https://orcid.org/0000-0002-6205-5408

Deleersnijder E

Delhey K https://orcid.org/0000-0001-5190-5406

DeLong J https://orcid.org/0000-0003-0558-8213

Denlinger D https://orcid.org/0000-0002-6781-4581

Denny M

DeRango E

Derégnaucourt S

Dermauw W https://orcid.org/0000-0003-4612-8969

Derry A

Deryk T

Desjonquères C https://orcid.org/0000-0002-6150-3264

Devaud J-M https://orcid.org/0000-0002-9873-2204

Deviche P

Devigili A https://orcid.org/0000-0001-8104-5195

Dexter K

Di Plinio S

Diamant E https://orcid.org/0000-0003-1727-5855

Dias RA

Dias VS https://orcid.org/0000-0001-9904-2849

Didham R

Dillon EM. https://orcid.org/0000-0003-0249-027X

Dineen A

Dingle C https://orcid.org/0000-0001-9176-9787

Dixit N https://orcid.org/0000-0002-2145-9828

Dolotovskaya S https://orcid.org/0000-0002-1401-3764

Dominy N https://orcid.org/0000-0001-5916-418X

Doña J https://orcid.org/0000-0002-5075-9627

Donahue M https://orcid.org/0000-0002-7529-001X

Donelson J https://orcid.org/0000-0002-0039-5300

DONG Y https://orcid.org/0000-0003-4550-2322

Donini A

Donnelly R https://orcid.org/0000-0001-7642-0317

Donoghue P https://orcid.org/0000-0003-3116-7463

Donovan B

Dores Cruz T https://orcid.org/0000-0002-4792-8469

Doris G https://orcid.org/0000-0002-9144-3426

Dorn N

Dorrough A

dos Reis M

Doube M https://orcid.org/0000-0002-8021-8127

Dougherty L https://orcid.org/0000-0003-1406-0680

Doughty C

Douglas R

Downie J

Downing P https://orcid.org/0000-0002-5286-3153

Downs C

Dr. Camilla Sr

Dreissig S https://orcid.org/0000-0002-4766-9698

Dridi S

Drijfhout F

Driscoll D

Drobniak S

Dubé C https://orcid.org/0000-0002-8990-8597

Dubois K

Dudash M

Dudley R https://orcid.org/0000-0003-3707-5682

Dueñas L https://orcid.org/0000-0002-0756-826X

Dukas R https://orcid.org/0000-0003-4533-8542

Duneau D https://orcid.org/0000-0002-8323-1511

Dunhill A https://orcid.org/0000-0002-8680-9163

Dunn F

Durant A

Duriez O https://orcid.org/0000-0003-1868-9750

Durso A https://orcid.org/0000-0002-3008-7763

Dusek A https://orcid.org/0000-0001-8552-4089

Dutoit L

DuVal E

Dziuba M https://orcid.org/0000-0003-1803-4164

Eastwood J https://orcid.org/0000-0002-5294-3321

Edes A

Edger P

Edgington M https://orcid.org/0000-0003-1922-9529

Eggleton P https://orcid.org/0000-0002-1420-7518

Ehlman S https://orcid.org/0000-0001-6981-9549

Ehrlen J https://orcid.org/0000-0001-8539-8967

Eisen K

Eisenberg D https://orcid.org/0000-0003-0812-1862

Eiting T

Elias D https://orcid.org/0000-0002-5895-4275

Elias M

Elias S

Elizabeth B

Elkrewi M https://orcid.org/0000-0002-5328-7231

Ellen J

Elling J

Ellis S https://orcid.org/0000-0001-9019-6040

Ellner S https://orcid.org/0000-0002-8351-9734

Emily B

Emlet R

Emma R

Endler J https://orcid.org/0000-0002-7557-7627

Engert V

Ensminger D https://orcid.org/0000-0001-5554-1638

Eppinga M https://orcid.org/0000-0002-1954-6324

Erwin D https://orcid.org/0000-0003-2518-5614

Esbaugh A https://orcid.org/0000-0002-7262-4408

Esmaeili S

Estévez-Barcia D https://orcid.org/0000-0002-5852-8145

Estrada-Peña A

Evans A

Evans K

Evans S https://orcid.org/0000-0001-5812-4039

Evers S https://orcid.org/0000-0002-2393-5621

E-Vojtkó A

Evy van B

Eyster H https://orcid.org/0000-0002-5571-3126

Eziz A

Fagan W https://orcid.org/0000-0003-2433-9052

Faivovich J

Falk J

Falkingham P

Fallon A

Fan P-F https://orcid.org/0000-0003-4747-5727

Farhat E

Fariña R https://orcid.org/0000-0003-0898-0333

Farine D https://orcid.org/0000-0003-2208-7613

Farmer D'J

Farrell M https://orcid.org/0000-0003-0452-6993

Farris D https://orcid.org/0000-0002-6720-1961

Fauteux D https://orcid.org/0000-0001-5373-8701

Fawcett T https://orcid.org/0000-0001-6337-901X

Fayle T

Fedak M

Federico MG

Feller KD

Feng P https://orcid.org/0000-0002-6742-4938

Fenton A https://orcid.org/0000-0002-7676-917X

Fenton B

Fernandez J

Fernandez-Betelu O https://orcid.org/0000-0002-6002-4376

Ferreira S

Ferrier D

Ferrigno S

Fetter-Pruneda I

Fieder M https://orcid.org/0000-0002-3257-8902

Fiedler K

Fiedler W

Figueirido Castillo FB https://orcid.org/0000-0003-2542-3977

Figuerola J

Filippa E

Filippi P https://orcid.org/0000-0002-2990-9344

Fiorini V https://orcid.org/0000-0003-0447-461X

Fischer A https://orcid.org/0000-0001-5922-8411

Fischer E https://orcid.org/0000-0002-2916-0900

Fischer J https://orcid.org/0000-0002-5807-0074

Fisher A https://orcid.org/0000-0002-9532-9575

Fisher B https://orcid.org/0000-0002-4653-3270

Fisher H https://orcid.org/0000-0001-5622-4335

Fitton L

Fitzgibbon L https://orcid.org/0000-0002-8563-391X

Fitzpatrick L https://orcid.org/0000-0003-3390-2070

Fitzpatrick S

Fleishman L https://orcid.org/0000-0002-2309-7496

Fleming R https://orcid.org/0000-0001-5033-5069

Flintham E https://orcid.org/0000-0002-8037-5560

Floeder S

Florian L

Font E

Foo YZ https://orcid.org/0000-0001-7627-2991

Forbes A https://orcid.org/0000-0001-8332-6652

Forbes S https://orcid.org/0000-0001-9064-9409

Fornaciai M

Forrest J

Forstmeier W https://orcid.org/0000-0002-5984-8925

Foster S https://orcid.org/0000-0003-4760-8390

Fostowicz-Frelik L https://orcid.org/0000-0002-1266-1178

Fountain-Jones N https://orcid.org/0000-0001-9248-8493

Fowler M

Fowler N https://orcid.org/0000-0002-7355-6347

Fox C https://orcid.org/0000-0002-7545-7967

Fox L https://orcid.org/0000-0001-6550-7142

Fraimout A https://orcid.org/0000-0003-4552-3553

Francis AL

Francisco F

Frankish C

Franks S

Frei D

Freitas R

Frenoy A https://orcid.org/0000-0002-3539-8915

Frentiu FD https://orcid.org/0000-0001-8628-4216

Frere C https://orcid.org/0000-0002-9671-2138

Friant S https://orcid.org/0000-0003-1664-5180

Friberg U https://orcid.org/0000-0001-6112-9586

Fricke E http://orcid.org/0000-0002-0520-4200

Friedlander T

Friedman M https://orcid.org/0000-0002-0114-7384

Friesen C https://orcid.org/0000-0001-5338-7454

Friman V

Friston K https://orcid.org/0000-0001-7984-8909

Frith C

Fröbisch N

Fromm B

Frommen J https://orcid.org/0000-0002-1752-6944

Frost C

Fruciano C

Fu F

Fu S-J https://orcid.org/0000-0001-7665-9037

Fujisawa T

Fujiwara S-i https://orcid.org/0000-0001-9393-5734

Fusi M

Fuxjager M https://orcid.org/0000-0003-0591-6854

Gabay S

Gabor C https://orcid.org/0000-0001-7584-1451

Gaboriau T

Gabriela Š https://orcid.org/0000-0002-4698-6405

Gabrielle W

Gaeta G

Gagen M

Gagne S

Gaillard J-M

Gaines R https://orcid.org/0000-0002-3713-5764

Gaitan-Espitia J https://orcid.org/0000-0001-8781-5736

Galen S https://orcid.org/0000-0003-0209-1535

Gall M

Gallardo B

Gamboa M

Ganel T

García-Callejas D https://orcid.org/0000-0001-6982-476X

García-Peña G https://orcid.org/0000-0002-3515-6908

Gardner A https://orcid.org/0000-0001-8438-0634

Gardner A https://orcid.org/0000-0002-1304-3734

Garg K https://orcid.org/0000-0003-0915-7314

Garlovsky M https://orcid.org/0000-0002-3426-4341

Garroway C https://orcid.org/0000-0002-0955-0688

Gaskell J https://orcid.org/0000-0001-9583-323X

Gasparini C https://orcid.org/0000-0001-9172-1142

Gatti J-L https://orcid.org/0000-0001-7683-718X

Gaudry Q

Gauzere J

Gavin M https://orcid.org/0000-0002-2169-4668

Gaynor M https://orcid.org/0000-0002-3912-6079

Gearty W https://orcid.org/0000-0003-0076-3262

Geber M

George EA

Geppert C https://orcid.org/0000-0003-1970-4131

Gerber L https://orcid.org/0000-0002-1247-2262

Gerdol M

Germain R https://orcid.org/0000-0002-1270-6639

Gerth M https://orcid.org/0000-0001-7553-4072

Gervais L

Gervais M

Ghalambor CK

Ghanim M

Ghosh M

Giaimo S https://orcid.org/0000-0003-0421-3065

Gibbs L https://orcid.org/0000-0001-7461-3393

Gibert J https://orcid.org/0000-0002-5083-6418

Gibson A https://orcid.org/0000-0002-0867-4953

Gibson B

Giezendanner J

Gil R https://orcid.org/0000-0002-9397-1003

Gilad-Gutnick S https://orcid.org/0000-0001-5826-1674

Gilbert F

Gilchrist M

Gillies N https://orcid.org/0000-0002-9950-609X

Gillingham M https://orcid.org/0000-0002-7935-9539

Gilman R https://orcid.org/0000-0003-2000-7966

Gilmour K

Giomi F https://orcid.org/0000-0002-7624-9457

Girard F https://orcid.org/0000-0002-7846-2710

Giribet G https://orcid.org/0000-0002-5467-8429

Gloag R https://orcid.org/0000-0002-2037-4267

Glykou A

Godbold J

Godin J-G http://orcid.org/0000-0001-8465-1721

Gogarten JP

Gogarten J https://orcid.org/0000-0003-1889-4113

Golbuu Y

Goldman A https://orcid.org/0000-0003-1115-3697

Goller F https://orcid.org/0000-0001-5333-1987

Gols R https://orcid.org/0000-0002-6839-8225

Gomes D https://orcid.org/0000-0002-2642-3728

Goncalves Souza T

Gonzalez-Castro A

González-Lagos C

Gonzalez-Tokman D https://orcid.org/0000-0001-7251-5773

Gonzalez-Voyer A

Goodisman M https://orcid.org/0000-0002-4842-3956

Goos M

Gosliner T

Gotthard K

Götzenberger L https://orcid.org/0000-0003-3040-2900

Goupil L

Govaert L https://orcid.org/0000-0001-8326-3591

Gownari N

Grace H

Graham K https://orcid.org/0000-0002-7422-7676

Graham L

Gray M

Grecian WJ

Green D

Greenberg D https://orcid.org/0000-0001-7489-1393

Greenfield M https://orcid.org/0000-0003-1935-3423

Gregoire A

Gregory R https://orcid.org/0000-0002-7419-5053

Greiner A

Greischar M https://orcid.org/0000-0002-7521-9344

Greives T

Grether G https://orcid.org/0000-0003-3441-0537

Greyson C

Griebel A

Griesser M

Griffin A

Griffin J

Griffith S

Griffiths H

Griffiths H

Griffiths J https://orcid.org/0000-0003-0319-515X

Grogan K

Groom H

Groothuis T https://orcid.org/0000-0003-0741-2174

Grosjean P https://orcid.org/0000-0002-3623-8083

Grossnickle D https://orcid.org/0000-0002-8825-2186

Grossnickle DM

Gruber-Vodicka H

Grueter C https://orcid.org/0000-0001-8770-8148

Grunst M https://orcid.org/0000-0002-3425-4020

Gruntman M https://orcid.org/0000-0003-2228-9266

Guadalupe Sepulveda ZM

Guariento R https://orcid.org/0000-0003-2035-2030

Guerra-Grenier E

Guglielmo C

Guiguen Y https://orcid.org/0000-0001-5464-6219

Guillaume F

Guillon Q

Gulia-Nuss M

Gumbs R

Guo B https://orcid.org/0000-0002-9796-5700

Gupta S

Guschanski K

Gustison M

Gutak J

Guzman LM

Gweon H https://orcid.org/0000-0001-8012-3526

Hadfield J

Haesevoets T

Häfker S

Hahn MW

Hahn P

Hailey C

Hakala S

Halali S https://orcid.org/0000-0002-9960-3682

Halfwerk W https://orcid.org/0000-0002-4111-0930

Hall D

Hall M https://orcid.org/0000-0002-4738-203X

Hämäläinen A https://orcid.org/0000-0001-9260-8299

Hamann E

Hamel S

Hamelin F

Hamid R

Hamilton D https://orcid.org/0000-0001-5883-0136

Hammerschmidt K

Han C https://orcid.org/0000-0001-9261-4031

Hanke F https://orcid.org/0000-0002-1737-3861

Hanley D https://orcid.org/0000-0003-0523-4335

Hannah G https://orcid.org/0000-0003-3550-3890

Hansen J https://orcid.org/0000-0001-5795-1761

Hansol Ryu

Hansson I

Harcombe W

Hare M

Hareli S

Harman J https://orcid.org/0000-0002-1563-4917

Harms K

Harper D

Harper E https://orcid.org/0000-0002-1092-3867

Harrigan R

Harrison J https://orcid.org/0000-0001-5223-216X

Harrison R

Harshavardhan T

Hart B https://orcid.org/0000-0003-2342-6058

Hart N

Hartfield M

Hartley I https://orcid.org/0000-0002-7592-3921

Harvell D

Harvey C https://orcid.org/0000-0002-2830-0844

Harvey E http://orcid.org/0000-0002-8601-7326

Harvey M

Harzsch S https://orcid.org/0000-0002-8645-3320

Hassall C

Hassett B

Hatala K https://orcid.org/0000-0001-9131-5304

Hatchwell B

Hattich G

Havird J https://orcid.org/0000-0002-8692-6503

Hawlena H https://orcid.org/0000-0002-0634-2920

Hawley D https://orcid.org/0000-0001-9573-2914

Hayward M W. https://orcid.org/0000-0002-5574-1653

Hazzi N

Heard S https://orcid.org/0000-0002-5976-1133

Hedenström A https://orcid.org/0000-0002-1757-0945

Heidinger B https://orcid.org/0000-0003-0064-209X

Heitlinger E

Heldmaier G

Hellmann J https://orcid.org/0000-0003-0479-3960

Hemingson C https://orcid.org/0000-0002-7127-6288

Hemmi J https://orcid.org/0000-0003-4629-9362

Henrichs D

Henrique Galante TW

Henry Y https://orcid.org/0000-0001-5972-9136

Herman J https://orcid.org/0000-0003-4104-2322

Herrera-Alsina L https://orcid.org/0000-0003-0474-3592

Hilbe C

Hilborn R

Hill E https://orcid.org/0000-0002-2992-2004

Hill G

Hill P https://orcid.org/0000-0002-6190-6426

Hill R

Hill T https://orcid.org/0000-0003-4159-9104

Hillyer J https://orcid.org/0000-0002-3178-0201

Hiltunen T https://orcid.org/0000-0001-7206-2399

Hines J

Hirt M https://orcid.org/0000-0002-8112-2020

Hladish T

Hobson K

Hochstrasser M

Hofer M

Hoffmann A https://orcid.org/0000-0001-9497-7645

Holland D

Holland S

Holman L https://orcid.org/0000-0002-7268-2173

Holmes J https://orcid.org/0000-0001-8804-2149

Holmes M

Holovachov O https://orcid.org/0000-0002-4285-0754

Holowka N https://orcid.org/0000-0003-0593-7524

Holt N

Holtmann B https://orcid.org/0000-0002-2995-7274

Homeier J

Homma K

Hong X-Y https://orcid.org/0000-0002-5209-3961

Hood W https://orcid.org/0000-0002-8398-3908

Hooper B

Hoover K

Hopkins B https://orcid.org/0000-0002-9760-6185

Hopkins MJ https://orcid.org/0000-0002-3580-2172

Houle D

Houslay T https://orcid.org/0000-0001-5592-9034

Hoverman J

Hovestadt T https://orcid.org/0000-0001-7368-6013

Howard L

Hoye B

Hsieh C-H

Hu J https://orcid.org/0000-0001-9607-1863

Hu J

Hu Y

Hua F http://orcid.org/0000-0001-8514-8757

Huang H-T

Huang Y-H https://orcid.org/0000-0002-2961-0895

Huey R https://orcid.org/0000-0002-4962-8670

Hughes AR

Hughes B

Hulber K

Hund A

Hunt G https://orcid.org/0000-0001-6430-5020

Hunting E https://orcid.org/0000-0002-8794-3452

Hurlbert A

Hurst G

Hussain A

Hut R https://orcid.org/0000-0003-4552-0985

Hyland Bruno J

Iannarilli F https://orcid.org/0000-0002-7018-3557

Iijima M https://orcid.org/0000-0003-2701-0391

Ilany A https://orcid.org/0000-0001-8089-569X

Imogene W

Ingels J

Inouye B

Inouye D

Iossa G https://orcid.org/0000-0001-6813-4361

Irina P https://orcid.org/0000-0001-8854-2637

Iritani R https://orcid.org/0000-0002-2396-1109

Irvine K

Isabella L https://orcid.org/0000-0003-4674-7751

Isaksson C https://orcid.org/0000-0002-6889-1386

Ito T

Ivry R

Iwaniuk A https://orcid.org/0000-0001-9273-3655

Jaakkola K https://orcid.org/0000-0002-4113-748X

Jablonski D

Jack R

Jackendoff R

Jackson M

Jacob S

Jacobs A

Jacobsen M

Jakob H https://orcid.org/0000-0001-5795-1761

Jakub Zak https://orcid.org/0000-0003-2845-8323

Jambura PL https://orcid.org/0000-0002-4765-5042

James H

James T

Janis C

Jankauski M https://orcid.org/0000-0001-6305-0564

Jannik M

Jansson F https://orcid.org/0000-0001-8357-0276

Januchowski-Hartley F https://orcid.org/0000-0003-2468-8199

Jarochowska E https://orcid.org/0000-0001-8937-9405

Jay Gallagher

Jeff Girard

Jenner R

Jenni-Eiermann S

Jensen F https://orcid.org/0000-0001-8776-3606

Jerilyn C

Jessica G

Jessica K

Jesus Hernandez M

Ji Y

Jiang L

Jian-Li Z https://orcid.org/0000-0002-5137-7735

Jia-Syuan C https://orcid.org/0000-0002-6587-9197

Jiggins F https://orcid.org/0000-0001-7470-8157

Joanna C https://orcid.org/0000-0001-7613-8127

Jockusch E https://orcid.org/0000-0003-4718-0531

Joel H

Joëlle J

Johnsen A

Johnson D https://orcid.org/0000-0003-2299-2525

Johnson T

Johnson-Ulrich L https://orcid.org/0000-0001-5604-5935

Johnston A

Johnston R

Jon R

Jones H https://orcid.org/0000-0003-1402-4778

Jones M https://orcid.org/0000-0002-4469-7896

Jones W https://orcid.org/0000-0002-3058-0072

Jonsson T https://orcid.org/0000-0002-5049-7612

Jordan L

Jorge SG

Jørgensen F

Jørgensen L

Jorrit Lucas

Jose Miguel P https://orcid.org/0000-0001-8457-7840

Josefina L https://orcid.org/0000-0002-1283-2491

Jost B

Jueterbock A

Julián CV

Juliana J

Jupiter S

Kaaronen R

Kacelnik A

Kafer J https://orcid.org/0000-0002-0561-8008

Kaiser D https://orcid.org/0000-0002-9007-3160

Kaiser S

Kalinkat G https://orcid.org/0000-0003-3529-5681

Kaliontzopoulou A https://orcid.org/0000-0002-7897-7204

Kallio E https://orcid.org/0000-0003-2991-612X

Kaltz O

Kamp-Becker I

Kandylaki KD

Kane A https://orcid.org/0000-0002-2830-5338

Kang C https://orcid.org/0000-0003-3707-4989

Kanngiesser P https://orcid.org/0000-0003-1068-3725

Kano F https://orcid.org/0000-0003-4534-6630

Kaplan J

Karamon J

Karban R https://orcid.org/0000-0002-1565-2429

Kardos M https://orcid.org/0000-0002-7587-3004

Karin HO https://orcid.org/0000-0002-1695-0989

Karp D https://orcid.org/0000-0002-3832-4428

Karri N

Kate L

Kate MB https://orcid.org/0000-0002-1876-3132

Katharine S

Katherine M https://orcid.org/0000-0002-0795-3096

Kathleen G

Katja K

Katsuki M

Kaufmann J

Kaufmann P

Kaur R

Kearney M

Kefford B http://orcid.org/0000-0001-6789-4254

Keisha B https://orcid.org/0000-0002-8092-833X

Kekäläinen J https://orcid.org/0000-0001-6303-6797

Kelber A https://orcid.org/0000-0003-3937-2808

Keller L

Kelly C https://orcid.org/0000-0002-0693-7211

Kelly M https://orcid.org/0000-0001-6998-5053

Kemppainen P

Kendall W

Kenkel C

Kennedy D https://orcid.org/0000-0003-0820-115X

Kennedy P https://orcid.org/0000-0002-2524-6192

Kershenbaum A

Kerth G

Khelifa R https://orcid.org/0000-0001-6632-8787

Kibbe M

Kiguchi N

Kikuchi D https://orcid.org/0000-0002-7379-2788

Kilpatrick AM https://orcid.org/0000-0002-3612-5775

Kim H

Kim S-Y https://orcid.org/0000-0002-5170-8477

Kim T

Kindsvater H

King B

King W

Kingsolver J https://orcid.org/0000-0002-9839-0208

Kirkman K

Kisdi É https://orcid.org/0000-0002-7096-4222

Kitchener A https://orcid.org/0000-0003-2594-0827

Kiyatkin E

Kjernsmo K https://orcid.org/0000-0002-8570-0406

Klatzky R

Klauschies T

Klenke R

Klinger E

Klug H https://orcid.org/0000-0003-3169-2452

Knight T

Kocot K https://orcid.org/0000-0002-8673-2688

Koenig W https://orcid.org/0000-0001-6207-1427

Kohl K https://orcid.org/0000-0003-1126-2949

Kokko H https://orcid.org/0000-0002-5772-4881

Komata S https://orcid.org/0000-0001-7231-6344

Komdeur J https://orcid.org/0000-0002-9241-0124

Konar B

Kondoh M https://orcid.org/0000-0001-5779-9392

Koonin E https://orcid.org/0000-0003-3943-8299

Koop JAH

Kooyers N https://orcid.org/0000-0003-3398-7377

Korin J

Koski M https://orcid.org/0000-0002-0274-3145

Koteja P https://orcid.org/0000-0003-0077-4957

Kotrschal A https://orcid.org/0000-0003-3473-1402

Koyabu D https://orcid.org/0000-0002-4087-7742

Kozłowski J https://orcid.org/0000-0002-7084-2030

Kramer N https://orcid.org/0000-0001-5150-0732

Krams I https://orcid.org/0000-0001-7150-4108

Kratochvil L https://orcid.org/0000-0002-3515-729X

Kreiner J

Krems J https://orcid.org/0000-0002-2590-2241

Kret M https://orcid.org/0000-0002-3197-5084

Krishnadas M

Kristensen J

Kristensen T

Kristiana B

Kristin R

Krivan V https://orcid.org/0000-0003-0971-1231

Kröel-Dulay G

Kroumi D https://orcid.org/0000-0003-4861-0361

Krueger F

Krumhuber E

Krupenye C https://orcid.org/0000-0003-2029-1872

Krupp D

Kudo G https://orcid.org/0000-0002-6488-818X

Kuehn S

Kuhn J

Kulahci I https://orcid.org/0000-0003-0104-0365

Kurosawa G

Kurschilgen M

Kurvers R https://orcid.org/0000-0002-3460-0392

Kutsukake N https://orcid.org/0000-0001-8637-6233

Kuyper T

Kyogoku D https://orcid.org/0000-0002-6214-8241

Laanto E

Labonte D https://orcid.org/0000-0002-1952-8732

Lafferty K https://orcid.org/0000-0001-7583-4593

Lahdenperä M

Lamaze A

Lambert O

Lambrechts L https://orcid.org/0000-0001-5958-2138

Lameira A https://orcid.org/0000-0003-3071-4824

Lamichhaney S

Lamont T

Landi P https://orcid.org/0000-0002-6373-2992

Lane S https://orcid.org/0000-0002-3797-3178

Langergraber K

Langley E

Lanlan C

Lanta V

Laporta G

Lappin AK https://orcid.org/0000-0002-5386-0069

Laris P

Larremore D

Lattorff M https://orcid.org/0000-0002-8603-6332

Latzel V

Lau P

Laura JJ

Laura G https://orcid.org/0000-0002-7636-4818

Lautenschlager S https://orcid.org/0000-0003-3472-814X

Laviale M https://orcid.org/0000-0002-9719-7158

Law C https://orcid.org/0000-0003-1575-7746

Lawson C

Lawton C

Lazzaro B https://orcid.org/0000-0002-3881-0995

Le Provost G

Leache A https://orcid.org/0000-0001-8929-6300

Leahy L

Leal CG

Leavens D

Leavitt P https://orcid.org/0000-0001-9805-9307

Lebarbenchon C https://orcid.org/0000-0002-0922-7573

Lebenzon J

Leblanc A

Leclere L

Ledda A https://orcid.org/0000-0002-2387-3774

Lee J

Lee KP https://orcid.org/0000-0001-9770-0078

Lees A

Legendre F

Lehmann J

Lehmann L https://orcid.org/0000-0001-9549-9577

Lehnert M https://orcid.org/0000-0003-1999-196X

Lehtinen S https://orcid.org/0000-0002-4236-828X

Leighton G https://orcid.org/0000-0003-0564-3980

Leinwand J

Leith N https://orcid.org/0000-0002-5267-9014

Leitner S https://orcid.org/0000-0002-9482-0362

Lello J http://orcid.org/0000-0002-2640-1027

Lemaitre J-F https://orcid.org/0000-0001-9898-2353

Lennon J https://orcid.org/0000-0003-3126-6111

Lent D

Léo C https://orcid.org/0000-0001-6914-7973

Leroux M https://orcid.org/0000-0001-7087-449X

Leung J

Levesque D https://orcid.org/0000-0003-0132-8094

Levi T

Levin L https://orcid.org/0000-0002-2858-8622

Levin M https://orcid.org/0000-0001-7292-8084

Levis N https://orcid.org/0000-0001-7650-2371

Levy O

Lewanzik D

Lewis L https://orcid.org/0000-0003-1693-5065

Lhoraynne Pereira G https://orcid.org/0000-0002-8450-207X

Li P https://orcid.org/0000-0001-9481-1282

Li Q-J

Li R https://orcid.org/0000-0001-9601-6020

Li Y https://orcid.org/0000-0001-9080-507X

Li Y

Liao J https://orcid.org/0000-0002-9520-3235

Liberti J

Librado P

Lichtenberg E

Liedvogel M https://orcid.org/0000-0002-8372-8560

Liénard MA https://orcid.org/0000-0003-3193-3666

Lifjeld JT

Liker A

Lim H

Lim J

Limmathurotsakul D https://orcid.org/0000-0001-7240-5320

Lin S

Lin Y-C

Lind O

Lindecke O https://orcid.org/0000-0002-2545-9999

Lindholm A https://orcid.org/0000-0001-8460-9769

Lindsay R https://orcid.org/0000-0003-2063-9285

Lindstedt C https://orcid.org/0000-0001-8176-3613

Link A

Linnen C

Liow LH

Lipshutz S https://orcid.org/0000-0002-9816-2977

Lisi M https://orcid.org/0000-0003-3554-385X

Lisovski S

Liu H https://orcid.org/0000-0002-8687-3237

Liu O https://orcid.org/0000-0002-9735-3384

Liu Y https://orcid.org/0000-0003-4580-5518

Liu Y https://orcid.org/0000-0003-3948-1246

Liu Y https://orcid.org/0000-0002-2346-740X

Livia P https://orcid.org/0000-0002-1013-039X

Lloyd-Smith J

Lo N

Locatello L https://orcid.org/0000-0002-3530-2565

Loe LE

Löfstedt C

Logan C

London S http://orcid.org/0000-0002-7839-2644

Long RA

Longdon B https://orcid.org/0000-0001-6936-1697

Longo M https://orcid.org/0000-0002-2450-4903

Longrich N https://orcid.org/0000-0002-9586-8907

Lopez-Uribe MM https://orcid.org/0000-0002-8185-2904

Lord J http://orcid.org/0000-0001-6616-1526

Lorenzo M

Loughran C

Lowther A https://orcid.org/0000-0003-4294-3157

Luis M

Luis A

Lukas D https://orcid.org/0000-0002-7141-3545

Luo S

Luo Y-J https://orcid.org/0000-0002-3418-3146

Luther D https://orcid.org/0000-0003-3331-6186

Lycett S https://orcid.org/0000-0003-3159-596X

Lymbery R https://orcid.org/0000-0001-8041-6169

Lynch V

Ma L

Ma W-J

Maas M https://orcid.org/0000-0003-0122-106X

Macartney E https://orcid.org/0000-0003-3866-143X

MacColl A https://orcid.org/0000-0003-2102-6130

MacDougall-Shackleton E https://orcid.org/0000-0002-4138-6398

MacIntosh A

Mackay T

MacKenzie B

MacLean H https://orcid.org/0000-0003-0982-3209

MacMahon D

Maggi E https://orcid.org/0000-0001-6934-9380

Magierecka A https://orcid.org/0000-0003-0873-6904

Maglieri V https://orcid.org/0000-0001-6631-043X

Magnhagen C

Maher S https://orcid.org/0000-0002-3430-0410

Maier S

Majer J

Makowski D

Malani A

Mallo M

Mallott E https://orcid.org/0000-0001-5446-8563

Mammola S https://orcid.org/0000-0002-4471-9055

Manenti R

Manger P

Manikandan B

Manlick P

Manuel Fuertes R https://orcid.org/0000-0003-0736-8791

Marcellini S

Marcin D https://orcid.org/0000-0003-1803-4164

Marcondes R

Marean C

Margres M

Maria Colt https://orcid.org/0000-0002-3832-7100

Marianne M https://orcid.org/0000-0002-4861-3207

 Sara W

Clara K

Marieke de C

Mariette M https://orcid.org/0000-0003-0567-4111

Marion B

Marivaux L https://orcid.org/0000-0002-2882-0874

Marjakangas E-L https://orcid.org/0000-0002-5245-3779

Mark J https://orcid.org/0000-0002-3319-1443

Marko P

Marleau J

Marler C https://orcid.org/0000-0002-2783-1029

Marler CA

Marlétaz F

Márquez R

Marsh J

Marshall H

Marshall J

Marshall K https://orcid.org/0000-0002-6991-4957

Martin B

Martin G

Martin K

Martin RJ

Martínez-García R https://orcid.org/0000-0003-2765-8147

Martínez-Núñez C https://orcid.org/0000-0001-7814-4985

Masanori Y

Masese F https://orcid.org/0000-0002-5912-5049

Mashoodh R https://orcid.org/0000-0003-3065-9044

Mason G https://orcid.org/0000-0001-8045-4579

Mather J https://orcid.org/0000-0001-7009-1639

Mathilde S https://orcid.org/000030168192836

Mathot K https://orcid.org/0000-0003-2021-1369

Matsuo T https://orcid.org/0000-0002-4185-6740

Matsuzawa T https://orcid.org/0000-0002-8147-2725

Matthew T

Matthews B

Matthews B https://orcid.org/0000-0001-9089-704X

Matthews J http://orcid.org/0000-0002-2766-8671

Mattila H https://orcid.org/0000-0001-5712-1688

Matz M

Maxwell S

Mayer V https://orcid.org/0000-0001-6662-8237

McBride S https://orcid.org/0000-0001-5120-0115

McCabe B

McCabe L

McCallum E

McCann K

McCarthy M

McClelland G

McCulloch GA https://orcid.org/0000-0003-1462-7106

McCullough E https://orcid.org/0000-0002-6886-9445

McCurry M https://orcid.org/0000-0002-2452-1404

McElligott AG https://orcid.org/0000-0001-5770-4568

McFarland R https://orcid.org/0000-0001-8245-9269

McGaugh S https://orcid.org/0000-0003-3163-3436

McGee M

McGlothlin J https://orcid.org/0000-0003-3645-6264

McGrew W

McIlroy S https://orcid.org/0000-0003-2482-8817

McKee J

McKinnon J

McLeish M https://orcid.org/0000-0002-4525-5221

McMahon K

McNamara J https://orcid.org/0000-0002-4235-3045

McNeil J https://orcid.org/0000-0002-2702-0293

McPhee M

McPherson J

McWilliam M https://orcid.org/0000-0001-5748-0859

Md Moshiur R https://orcid.org/0000-0001-8319-395X

Meachen J

Medina I https://orcid.org/0000-0002-1021-5035

Medrano M

Meghan Z

Meineke EK

Melina S https://orcid.org/0000-0002-9651-9514

Mellin C

Mello M https://orcid.org/0000-0002-9098-9427

Mendenhall C

Mendl M https://orcid.org/0000-0002-5302-1871

Mendoza I

Menge B

Mennill D

Menon R

Mercedes Pascual MW

Merchant H

Meredith J

Merondun J

Merrill R

Merzendorfer H

Mescher M https://orcid.org/0000-0002-7908-3309

Metcalfe N https://orcid.org/0000-0002-1970-9349

Mettke-Hofmann C

Metz M

Meunier J https://orcid.org/0000-0001-6893-2064

Meyer JR

Meyer-Rochow VB https://orcid.org/0000-0003-1531-9244

Miatta M https://orcid.org/0000-0001-5288-730X

Michalska-Smith M https://orcid.org/0000-0002-0369-412X

Michelangeli M

Michelle H https://orcid.org/0000-0003-4257-3467

Millar J

Miller A

Miller C https://orcid.org/0000-0002-2534-2109

Miller E https://orcid.org/0000-0001-6856-3107

Miller-Struttmann N

Mills LS https://orcid.org/0000-0001-8771-509X

Minelli A https://orcid.org/0000-0003-3387-1489

Mineo P

Minias P https://orcid.org/0000-0002-7742-6750

Minich J

Mirosław Ś https://orcid.org/0000-0002-2311-5033

Mitchell E https://orcid.org/0000-0001-6517-2231

Mitchell L

Mitra C

Mitsutoshi T https://orcid.org/0000-0002-1254-1803

Miyamoto N

Miyashita T

Miyazaki R https://orcid.org/0000-0003-4067-8791

Mizumoto N https://orcid.org/0000-0002-6731-8684

Mo O

Modica MV

Moe B

Moerman F https://orcid.org/0000-0002-5164-0978

Mola J https://orcid.org/0000-0002-5394-9071

Moleón M https://orcid.org/0000-0002-3126-619X

Molina R

Molino de MS

Moll H

Moll R

Molleman F https://orcid.org/0000-0002-6551-266X

Molly B

Momigliano P

Monaco C

Monteiro A https://orcid.org/0000-0001-9696-459X

Monteiro T https://orcid.org/0000-0002-2836-8961

Montenegro M

Montiglio P-O

Montoya JM

Mooers A

Moore I

Moore J

Moore M

Moorter BV

Moradinour Z https://orcid.org/0000-0003-0203-8216

Morales-Castilla I https://orcid.org/0000-0002-8570-9312

Moran N https://orcid.org/0000-0003-2983-9769

Morash A

Moreira X

Moret Y https://orcid.org/0000-0002-2435-8416

Morgan T

Moriarty KM

Moriarty M

Morin EM https://orcid.org/0000-0002-4840-0156

Morran L https://orcid.org/0000-0002-6422-0374

Morris M https://orcid.org/0000-0001-9962-2982

Morsky B https://orcid.org/0000-0003-3608-1634

Mos B https://orcid.org/0000-0003-3687-516X

Moscatelli A https://orcid.org/0000-0001-6269-4536

Moscovice L

Moseby K https://orcid.org/0000-0003-0691-1625

Mosquera GF

Moura R https://orcid.org/0000-0002-7911-4734

Mourier J https://orcid.org/0000-0001-9019-1717

Moussian B

Mousumi G-H https://orcid.org/0000-0002-4086-1601

Moya C https://orcid.org/0000-0001-7100-9115

Muchhala N https://orcid.org/0000-0002-4423-5130

Muchowska K

Mueldner G

Mukai H

Mukamel R

Mukta W https://orcid.org/0000-0002-3367-3944

Müller T

Mullon C https://orcid.org/0000-0002-9875-4227

Munasinghe M

Munch S

Mungee M https://orcid.org/0000-0002-5452-7196

Mupepele A

Murdock C https://orcid.org/0000-0001-5966-1514

Murray G

Murray-Stoker D

Mutha P https://orcid.org/0000-0003-1617-4948

Nakano H

Nanninga G https://orcid.org/0000-0002-0134-1689

Narayan E

Nason P

Naug D https://orcid.org/0000-0001-9252-8037

Nave K

Neate-Clegg M https://orcid.org/0000-0001-9753-6765

Negishi J

Neil L Jr https://orcid.org/0000-0001-7912-1689

Nelson E https://orcid.org/0000-0003-0058-8409

Nelson W

Neogi U

Neubauer TA https://orcid.org/0000-0002-1398-9941

Neuschulz EL https://orcid.org/0000-0001-7526-2580

Newcombe N

Nichols J

Niedbalski S

Nietlisbach P https://orcid.org/0000-0002-6224-2246

Nieuwenhuis B https://orcid.org/0000-0001-8159-4784

Nikola D

Nilsson G https://orcid.org/0000-0002-3195-3053

Nilsson-Örtman V https://orcid.org/0000-0002-1491-4706

Nishitani S https://orcid.org/0000-0002-6234-295X

Noah L

Noble D https://orcid.org/0000-0001-9460-8743

Nogué S

Nolet B

Nord A https://orcid.org/0000-0001-6170-689X

Nord C

Norder S

Noreikiene K https://orcid.org/0000-0001-7529-4902

Norin T https://orcid.org/0000-0003-4323-7254

Norman K

Norris D https://orcid.org/0000-0003-4874-1425

Northfield T https://orcid.org/0000-0002-0563-485X

Nowicki S https://orcid.org/0000-0002-6564-905X

Nowlan N https://orcid.org/0000-0002-9083-6279

Nudds R https://orcid.org/0000-0002-7627-6324

Nugues M

Nussey D https://orcid.org/0000-0002-9985-0317

Nyamukondiwa C https://orcid.org/0000-0002-0395-4980

O'Connor J

O'Brien A https://orcid.org/0000-0002-8455-8620

Obrist D https://orcid.org/0000-0002-5645-6037

O'Connor EA

Odemer R https://orcid.org/0000-0003-2230-4294

O'Donnell S https://orcid.org/0000-0002-6338-7148

Ogden N https://orcid.org/0000-0002-1062-7283

Ohde T

Ohmer M https://orcid.org/0000-0002-5937-6585

Olea P P. https://orcid.org/0000-0001-5387-9598

Ollerton J

Olsson K https://orcid.org/0000-0002-1695-0989

Olsson M https://orcid.org/0000-0002-4130-1323

O'Neill M https://orcid.org/0000-0001-9614-7813

Orben R https://orcid.org/0000-0002-0802-407X

Orive M

Orkin J

Orozco-terWengel P https://orcid.org/0000-0002-7951-4148

Orr DJ

Ortega G https://orcid.org/0000-0001-5305-1467

Ortega-Hernández J https://orcid.org/0000-0002-6801-7373

Ortego F

Ortmann S

Oruc I

Osenberg C https://orcid.org/0000-0003-1918-7904

Osmond M

Osorio DC

Otto S https://orcid.org/0000-0003-3042-0818

Ovando D https://orcid.org/0000-0003-2120-7345

Overgaard J

Owen J https://orcid.org/0000-0003-1383-4816

Owens H https://orcid.org/0000-0003-0071-1745

P. Sebastián Tambusso https://orcid.org/0000-0001-7349-5292

Pacheco Coelho M Túlio https://orcid.org/0000-0002-7831-3053

Pacheco A https://orcid.org/0000-0002-7899-9657

Padian K https://orcid.org/0000-0002-1174-8431

Paez D https://orcid.org/0000-0001-9035-394X

Paitz R https://orcid.org/0000-0003-4609-4359

Palaoro A https://orcid.org/0000-0002-8629-0728

Palme R

Palmer AR

Pamenter M https://orcid.org/0000-0003-4035-9555

Pantea P

Pantel J

Panyushkina I

Papageorgiou D https://orcid.org/0000-0001-5535-6133

Papastamatiou Y https://orcid.org/0000-0002-6091-6841

Papoli H

Parker T

Parkos JJ

Parma A

Parodi F

Parrish C https://orcid.org/0000-0002-1836-6655

Parry L https://orcid.org/0000-0002-3910-0346

Pascalis O

Paskulin L

Patricia R https://orcid.org/0000-0001-6944-4134

Patrik W https://orcid.org/0000-0001-9931-2028

Paul B https://orcid.org/0000-0002-8927-1899

Paxton R

Peacock M https://orcid.org/0000-0001-9273-8464

Pearse D

Pearson H

Peay K

Pedersen A

Pedruzzi L

Pei-Chi H https://orcid.org/0000-0001-8896-8347

Pelisson D

Pelletier F

Pen I

Peña J https://orcid.org/0000-0002-4137-1823

Penalba J https://orcid.org/0000-0001-6549-6885

Perc M https://orcid.org/0000-0002-3087-541X

Pereira Lopes R

Perez Navarro M

Pérez-Asensio JN https://orcid.org/0000-0002-1393-7412

Perez-Barrales R

Perez-Lamarque B https://orcid.org/0000-0001-7112-7197

Perlman S

Perlmutter J

Perret C https://orcid.org/0000-0002-9180-0542

Pessarrodona A https://orcid.org/0000-0002-6057-9937

Petchey O

Peter Flood https://orcid.org/0000-0002-0772-4920

Peter BG

Petersen P

Petrou E https://orcid.org/0000-0001-7811-9288

Petrzelkova K

Petschenka G https://orcid.org/0000-0002-9639-3042

Pezner A

Pfeifer M https://orcid.org/0000-0002-6775-3141

Pfeiffer K

Pfenninger M

Phifer-Rixey M

Phillimore A https://orcid.org/0000-0002-6553-1553

Pick JL https://orcid.org/0000-0002-6295-3742

Pie M https://orcid.org/0000-0002-2949-4871

Piero C https://orcid.org/0000-0003-3378-2603

Pierre L https://orcid.org/0000-0002-9755-2949

Pierre G https://orcid.org/0000-0002-7529-3456

Pierrejean M

Piersma T

Pilastro A https://orcid.org/0000-0001-9803-6308

Pimiento C

Pires M

Pires MM https://orcid.org/0000-0003-2500-4748

Pirk C

Pirotta E https://orcid.org/0000-0003-3541-3676

Pitcher T

Plaistow S

Poblete J

Podos J

Pohnert G

Poinar H

Poissonier L-A

Pokorny T

Pomiankowski A https://orcid.org/0000-0002-5171-8755

Pompeu P

Pondeville E http://orcid.org/0000-0001-8545-8472

Pons J

Pons L

Porro L

Porter S

Portugal S https://orcid.org/0000-0002-2438-2352

Postma E https://orcid.org/0000-0003-0856-1294

Potter C https://orcid.org/0000-0002-5223-8112

Poulin R https://orcid.org/0000-0003-1390-1206

Powell S

Power S

Powers D https://orcid.org/0000-0003-1126-7141

Pravosudov V https://orcid.org/0000-0003-1117-7875

Price T https://orcid.org/0000-0002-4423-8999

Priede I https://orcid.org/0000-0002-5064-9751

Prieto-Torres D https://orcid.org/0000-0002-0493-5941

Prikryl T

Pröhl H

Proulx M

Ptacnik R

Pujol B

Pulido F https://orcid.org/0000-0002-2430-8080

Puneeth D https://orcid.org/0000-0001-6948-7089

Puniamoorthy N https://orcid.org/0000-0003-0651-8356

Purnell M

Putman N https://orcid.org/0000-0001-8485-7455

Puts D

Pyenson N https://orcid.org/0000-0003-4678-5782

Pyott S

Quesada A

Quinn B

Quinn J

Quinn K

Quintero I https://orcid.org/0000-0003-3844-0805

Rabajante J https://orcid.org/0000-0002-0655-0893

Rachael K

Radchuk V

Rädecker N https://orcid.org/0000-0002-2387-8567

Radeloff V

Radford C https://orcid.org/0000-0001-7949-9497

Raffard A https://orcid.org/0000-0003-0128-890X

Rahman M

Raia P https://orcid.org/0000-0002-4593-8006

Raichlen D

Raine N https://orcid.org/0000-0001-6343-2829

Rajan R https://orcid.org/0000-0002-7770-3711

Rajon E

Rakotomalala A

Rämä T https://orcid.org/0000-0001-8111-8075

Ramos J

Ramos R https://orcid.org/0000-0002-0551-8605

Ranganathan R

Rangel A

Ranke P https://orcid.org/0000-0003-3757-8626

Raoult V https://orcid.org/0000-0001-9459-111X

Rasanen K

Raven P https://orcid.org/0000-0002-8536-2646

Raverty S

Ravi S https://orcid.org/0000-0001-7397-9713

Rawlence N https://orcid.org/0000-0001-6968-6643

Raymond M

Reber S https://orcid.org/0000-0001-8015-2538

Rebollar EA

Rebolleda-Gomez M https://orcid.org/0000-0002-3592-4479

Reby D

Rechavi O https://orcid.org/0000-0001-6172-3024

Reddin C

Reddy S

Redmond M https://orcid.org/0000-0002-4657-7943

Reed E

Reed T

Reeve C https://orcid.org/0000-0002-0238-0575

Reichard M https://orcid.org/0000-0002-9306-0074

Reichert M https://orcid.org/0000-0002-0159-4387

Reichert S

Reid J

Reif J

Reimer J

Reinhold K

Remeš V

Renaudie J https://orcid.org/0000-0002-9107-1984

Restif O https://orcid.org/0000-0001-9158-853X

Revilla T

Reyne B https://orcid.org/0000-0002-4899-1240

Reynolds S https://orcid.org/0000-0001-5043-0242

Reznick D https://orcid.org/0000-0002-1144-0568

Ribera d'Alcalà M https://orcid.org/0000-0002-5492-9961

Richardson D https://orcid.org/0000-0001-7226-9074

Richter K

Ridley A https://orcid.org/0000-0001-5886-0992

Riede T https://orcid.org/0000-0001-6875-0017

Riesch R https://orcid.org/0000-0002-0223-1254

Riginos C

Rillo M https://orcid.org/0000-0002-2471-0002

Ringler E https://orcid.org/0000-0003-3273-6568

Rioual P

Rissler L

Ristevski J

Rito T

Ritson-Williams R

Rivaes R

Rivera-Cáceres KD

Rivest E https://orcid.org/0000-0002-8570-8379

Rizzoli A

Robert D

Roche B

Rodolfo-Metalpa R

Rodriguez Santiago M

Rodríguez C

Roessingh P

Roesti M

Rogers H

Rogers L https://orcid.org/0000-0002-9956-1769

Rohner P https://orcid.org/0000-0002-9840-1050

Rok J https://orcid.org/0000-0002-3506-5416

Rolff J https://orcid.org/0000-0002-1529-5409

Roll U

Rollinson N https://orcid.org/0000-0002-5091-8368

Romano V https://orcid.org/0000-0002-4128-9205

Ron F

Roos D

Root-Gutteridge H https://orcid.org/0000-0001-9854-2948

Roper M

Ros A https://orcid.org/0000-0003-3241-9722

Rosa P https://orcid.org/0000-0001-6944-4134

Rosario M https://orcid.org/0000-0001-9969-6746

Rosel P

Rosenheim J

Rosi E

Roslin T https://orcid.org/0000-0002-2957-4791

Rosner A

Rossina P

Rossion B https://orcid.org/0000-0002-1845-3935

Roswell M https://orcid.org/0000-0002-8479-9184

Rotella A

Rougemont Q

Roulston T

Rousseau J https://orcid.org/0000-0001-5797-3565

Roussel D https://orcid.org/0000-0002-8865-5428

Royle N

Roze D

Rubenstein D https://orcid.org/0000-0002-4999-3723

Rubio De Casas R http://orcid.org/0000-0003-4276-4968

Ruedenauer F https://orcid.org/0000-0002-2509-0554

Ruffley M

Ruiz-Gonzalez M

Ruktanonchai NW

Rundle H https://orcid.org/0000-0002-8288-8888

Rushworth C

Russell A

Ruther J https://orcid.org/0000-0002-1295-347X

Ryan R

Ryan M https://orcid.org/0000-0002-6381-9545

Ryan S https://orcid.org/0000-0002-4308-6321

Ryan S https://orcid.org/0000-0002-3105-7582

Ryo Y https://orcid.org/0000-0003-3545-7513

Ryu T

Saber E https://orcid.org/0000-0003-1920-1737

Sachell L

Sachs J https://orcid.org/0000-0002-0221-9247

Sadowska E https://orcid.org/0000-0003-1240-4814

Sæther B-E https://orcid.org/0000-0002-0049-9767

Sakata J https://orcid.org/0000-0003-2285-9310

Sakshi K

Salem H https://orcid.org/0000-0002-4135-7407

Salmón P https://orcid.org/0000-0001-9718-6611

Samuel A https://orcid.org/0000-0002-0779-9543

Samuni L

San Millan A https://orcid.org/0000-0001-8544-0387

Sanchez M https://orcid.org/0000-0001-7587-3648

Sandel B

Sandell L https://orcid.org/0000-0002-1716-9801

Sandkam B https://orcid.org/0000-0002-5043-9295

Sandstrom G

Sandtner W

Sanford E

Sansalone G

Santema P https://orcid.org/0000-0002-5765-3067

Santos F

Santure AW

Saran S https://orcid.org/0000-0002-0238-498X

Sarkies P

Sarremejane R

Sasha B https://orcid.org/0000-0002-7629-3642

Sato N https://orcid.org/0000-0001-5023-5375

Sato T https://orcid.org/0000-0002-4660-9669

Sauer E https://orcid.org/0000-0002-8339-6498

Savage C

Savitzky A

Savoca M

Sawicki G

Scanlan P https://orcid.org/0000-0002-6733-9653

Schachner E

Schaefer R

Schausberger P https://orcid.org/0000-0002-1529-3198

Schellenberg G https://orcid.org/0000-0003-3681-6020

Schenkel M https://orcid.org/0000-0002-7721-319X

Scheumann M https://orcid.org/0000-0002-4091-9500

Scheuring I

Schick R

Schiel D

Schielzeth H https://orcid.org/0000-0002-9124-2261

Schiffbauer J https://orcid.org/0000-0003-4726-0355

Schindler D

Schirmer A https://orcid.org/0000-0001-7163-9694

Schluter D https://orcid.org/0000-0003-1683-7836

Schmid L

Schmitt M

Schmitz O https://orcid.org/0000-0003-1515-2667

Schmoll T https://orcid.org/0000-0003-3234-7335

Schmucki R

Schnabel F https://orcid.org/0000-0001-8452-4001

Schoerning E

Schubert N https://orcid.org/0000-0001-6131-3543

Schülke O https://orcid.org/0000-0003-0028-9425

Schultheiss P https://orcid.org/0000-0002-6906-3507

Schultz J

Schurger A

Schütz A

Schweiger O

Schweikert L https://orcid.org/0000-0002-8639-3612

Scofield R https://orcid.org/0000-0002-7510-6980

Scott A

Secondi J https://orcid.org/0000-0001-8130-1195

Sedda L https://orcid.org/0000-0002-9271-6596

Seddon A https://orcid.org/0000-0002-8266-0947

Seebacher F https://orcid.org/0000-0002-2281-9311

Segall M https://orcid.org/0000-0002-4913-2106

Segar S https://orcid.org/0000-0001-6621-9409

Sekaquaptewa D

Selmoni O

Semmler R https://orcid.org/0000-0003-1257-5859

Senar JC https://orcid.org/0000-0001-9955-3892

Senner N

Seppälä O https://orcid.org/0000-0001-7902-3069

Serrano-Meneses MA

Sethi SA

Sewall B

Seyfarth R

Seymour C https://orcid.org/0000-0002-6729-2576

Seymour R https://orcid.org/0000-0002-3395-0059

Seymoure B

Sgolastra F https://orcid.org/0000-0002-2845-8297

Shafir S https://orcid.org/0000-0002-9068-1078

Shah A https://orcid.org/0000-0002-8454-7905

Shamberger K

Shaner P-J https://orcid.org/0000-0001-8112-4299

Shapiro B

Sharma P

Shaw L https://orcid.org/0000-0001-7332-0820

Sheehy-Skeffington J

Sheeran L

Shefelbine S

Shefferson R https://orcid.org/0000-0002-5234-3131

Shein-Idelson M https://orcid.org/0000-0001-8132-2826

Sheldon K https://orcid.org/0000-0002-3215-2223

Shelley S https://orcid.org/0000-0001-6628-0226

Shenk M https://orcid.org/0000-0003-2002-1469

Shi Z

Shibasaki S https://orcid.org/0000-0002-8196-0745

Shih CK

Shizuka D https://orcid.org/0000-0002-0478-6309

Shocket M https://orcid.org/0000-0002-8995-4446

Shrader A https://orcid.org/0000-0002-6451-6132

Shrestha U

Shriver R

Shubham R https://orcid.org/000-0002-7717-053X

Shuert C https://orcid.org/0000-0002-3202-4897

Shultz A

Shurin J

Sibert E https://orcid.org/0000-0003-0577-864X

Siebert T https://orcid.org/0000-0003-4090-5480

Sievers M

Sigeman H https://orcid.org/0000-0002-1457-4174

Sikkel P

Silk J

Silva B https://orcid.org/0000-0003-3877-1356

Simon C

Simons M

Sinclair B

Sindi S

Singh M https://orcid.org/0000-0002-6026-947X

Singhal S https://orcid.org/0000-0001-5407-5567

Siniscalchi M https://orcid.org/0000-0002-1592-6549

Sitters H

Sjöberg S

Skandalis D https://orcid.org/0000-0002-5126-9582

Sládeček M

Šlipogor V https://orcid.org/0000-0002-8842-4144

Sloan D https://orcid.org/0000-0002-3618-0897

Ślusarczyk M https://orcid.org/0000-0002-2311-5033

Smaldino P

Šmejkal M https://orcid.org/0000-0002-7887-6411

Smiley T https://orcid.org/0000-0001-5940-1755

Smith B

Smith D

Smith H

Smith M https://orcid.org/0000-0001-5660-1727

Smith T

Smith-Greenaway E

Snell-Rood E https://orcid.org/0000-0002-2972-5895

Snowdon C

Snyder R

Soderstrom K

Sofía Ten G https://orcid.org/0000-0002-7284-6916

Soha J

Sol D https://orcid.org/0000-0001-6346-6949

Soley F

Solieri L

Somanathan H https://orcid.org/0000-0002-4803-3894

Somero G

Sommer-Trembo C https://orcid.org/0000-0001-9952-2906

Somveille M https://orcid.org/0000-0002-6868-5080

Song C https://orcid.org/0000-0001-7490-8626

Song X https://orcid.org/0000-0002-3335-0029

Sonnenberg B https://orcid.org/0000-0003-4496-4269

Sørensen J

Sørensen M

Sorenson M

Soreq H https://orcid.org/0000-0002-0955-526X

Sorokowski P https://orcid.org/0000-0001-9225-9965

Souza AS

Spalding H

Spalink D https://orcid.org/0000-0003-3250-568X

Speijer D https://orcid.org/0000-0002-2340-2753

Speller C https://orcid.org/0000-0001-7128-9903

Spence J

Spezie G https://orcid.org/0000-0002-0590-6456

Spiers A

Spocter M https://orcid.org/0000-0003-1174-7444

Sprayberry JDH

Sprecher S https://orcid.org/0000-0001-9060-3750

Springborn M https://orcid.org/0000-0003-3476-8758

Srinivasan U https://orcid.org/0000-0002-8040-1124

Stabach J A.

Stachowicz J

Stager M https://orcid.org/0000-0002-5635-580X

Stallings C

Stamatakis A

Staňková K https://orcid.org/0000-0002-4519-0325

Stanley D https://orcid.org/0000-0001-8948-8409

Stansbury A

Starr T

Stawski C

Stearns S

Stein L https://orcid.org/0000-0002-4110-3562

Stelinski L

Stelkens R

Stepney S https://orcid.org/0000-0003-3146-5401

Stern D

Stevenson P https://orcid.org/0000-0002-0736-3619

Stevenson T https://orcid.org/0000-0003-2644-9685

Stewart A https://orcid.org/0000-0001-5234-3871

Stewart T https://orcid.org/0000-0002-7965-5901

Stien A https://orcid.org/0000-0001-8046-7337

Stier A https://orcid.org/0000-0002-5445-5524

Stinchcombe J

Storck-Tonon D

Stouffer P

Straka J

Strauch C

Strausfeld N

Streicher J https://orcid.org/0000-0002-3738-4162

Stritih-Peljhan N

Struck T

Stuart P

Stubbs T https://orcid.org/0000-0001-7358-1051

Stuessy T

Stumpner A

Sturdy C

Sucgang R

Sudyka J

Suggitt A

Sugiura S https://orcid.org/0000-0002-9655-2189

Šulc M https://orcid.org/0000-0002-7866-1795

Sullivan N

Summers K https://orcid.org/0000-0001-7292-4286

Sunagar K

Sundaram M https://orcid.org/0000-0003-4235-8783

Sunde J

Sundin J https://orcid.org/0000-0003-1853-4046

Suraci J

Sustaita D https://orcid.org/0000-0001-9932-909X

Suter P

Suz L

Suzuki T https://orcid.org/0000-0001-6405-7653

Suzuki Y

Svensson E https://orcid.org/0000-0001-9006-016X

Sweeny A https://orcid.org/0000-0003-4230-171X

Symons C https://orcid.org/0000-0003-4120-0327

Szabo B https://orcid.org/0000-0002-3226-8621

Szeska C

Szollosi G

Szorkovszky A https://orcid.org/0000-0001-9331-3214

Taborsky B https://orcid.org/0000-0003-1690-8155

Takamori K

Takemoto K https://orcid.org/0000-0002-6355-1366

Takeuchi T https://orcid.org/0000-0002-9916-8801

Tambussi C

Tamè L

Tanaka M https://orcid.org/0000-0003-2198-1402

Tanas J

Tanmay D https://orcid.org/0000-0001-5604-7965

Tatarnic N https://orcid.org/0000-0001-8641-4412

Taylor E

Taylor J

Tchernichovski O

Tebbich S

Teder T https://orcid.org/0000-0001-6587-9325

Temeles E https://orcid.org/0000-0002-8059-1474

Terrennce G https://orcid.org/0000-0002-3950-0243

Terry C

Teunissen N https://orcid.org/0000-0001-7028-0034

Thawley C https://orcid.org/0000-0002-6040-2613

Thazin Hy

Therrien F https://orcid.org/0000-0002-2652-908X

Thessen A

Thitaram C

Thomas BS https://orcid.org/0000-0002-5978-6912

Thomas F

Thomas M https://orcid.org/0000-0002-9353-0566

Thomas G https://orcid.org/0000-0002-1982-6051

Thomas S

Thompson Gonzalez N https://orcid.org/0000-0002-3195-1277

Thompson J

Thompson K https://orcid.org/0000-0002-1487-3254

Thompson L https://orcid.org/0000-0002-3911-1280

Thöni C https://orcid.org/0000-0003-1190-8471

Thorogood R https://orcid.org/0000-0001-5010-2177

Tidière M https://orcid.org/0000-0001-6339-5765

Tigano A

Tilman D

Ting C-Ti

Title P

Tiunov A

Tobias J https://orcid.org/0000-0003-2429-6179

Todorov O https://orcid.org/0000-0002-0295-7557

Tomášek O https://orcid.org/0000-0002-2657-5916

Tomasello M https://orcid.org/0000-0002-1649-088X

Tomioka K

Tong C http://orcid.org/0000-0001-5202-5507

Tonnabel J

Tordoni E

Torres Vanegas F

Torres-Dowdall J https://orcid.org/0000-0003-2729-6246

Tortuero E

Toth Z

Touchon J https://orcid.org/0000-0002-1643-348X

Toussaint S

Townsend S https://orcid.org/0000-0003-1504-1801

Traulsen A https://orcid.org/0000-0002-0669-5267

Travisano M https://orcid.org/0000-0001-8168-0842

Treberg J

Treidel L https://orcid.org/0000-0001-9390-8306

Tremblay L

Trevathan W

Trevisin C https://orcid.org/0000-0002-1576-6397

Triki Z https://orcid.org/0000-0001-5592-8963

Trinitapoli J

Trnka A

Tryjanowski P https://orcid.org/0000-0002-8358-0797

Tuci E

Tuljapurkar S

Tung J https://orcid.org/0000-0003-0416-2958

Turner A

Tusso S https://orcid.org/0000-0002-0612-9230

Tüzün N

Tyack P https://orcid.org/0000-0002-8409-4790

Tyler B

Tyler H

Tysklinid N

Tyson R https://orcid.org/0000-0002-7373-4473

Uecker H https://orcid.org/0000-0001-9435-2813

Ugo P https://orcid.org/0000-0002-3926-4607

Ujvari B https://orcid.org/0000-0003-2391-2988

Upchurch P

Urban M https://orcid.org/0000-0003-3962-4091

Uszko W

Valdés-Correcher E

Valente D https://orcid.org/0000-0001-6086-5135

Valentina A

Valenti-Silva A

Valido A

Valle D https://orcid.org/0000-0002-9830-8876

Valverde S https://orcid.org/0000-0002-2150-9610

van den Bogert T https://orcid.org/0000-0002-3791-3749

van der Zee M https://orcid.org/0000-0002-8728-8646

van Dorst R

Van Dyck H

van Hasselt S

van Houte S

Van Iten H

van Oers K

van Opstal F

Van Valkenburgh B

van Veen F

van Velzen E

Van Wassenbergh S https://orcid.org/0000-0001-5746-4621

Vandergast A

Vandvik V https://orcid.org/0000-0003-4651-4798

Vannatta T

Vannier J https://orcid.org/0000-0003-0998-1231

Varela S

Vargas A

Vavre F

Vázquez-Domínguez E https://orcid.org/0000-0001-6131-2014

Vedder O

Veilleux C

Veilleux H

Venkadesan M https://orcid.org/0000-0001-5754-7478

Venkataraman V https://orcid.org/0000-0001-5016-4423

Verberk W

Verbruggen F https://orcid.org/0000-0002-7958-0719

Verhulst E

Vervelde L

Verzijden M

Vijayakumar SP https://orcid.org/0000-0002-4210-2959

Villalobos F https://orcid.org/0000-0002-5230-2217

Villoutreix R https://orcid.org/0000-0002-1815-3844

Vincze O

Vinebrooke R

Vinn O https://orcid.org/0000-0002-2889-2544

Vinson J https://orcid.org/0000-0003-4319-6851

Virginia I-M https://orcid.org/0000-0003-3728-2622

Visser M https://orcid.org/0000-0002-1456-1939

Vitali A

Vitousek M https://orcid.org/0000-0003-2855-0832

Vivas M https://orcid.org/0000-0003-2712-417X

Voelker G https://orcid.org/0000-0003-3659-3971

Vogler A

Vogt K https://orcid.org/0000-0002-8763-342X

Voigt C https://orcid.org/0000-0002-0706-3974

von Lintig J

Vorobyev M https://orcid.org/0000-0001-7615-5816

Vun Wen J

Vuts J https://orcid.org/0000-0001-6240-0905

Wada H

Wade N

Waegele H

Wagner C

Wagner D

Wagner M

Wagner M

Wagner N https://orcid.org/0000-0001-9227-5674

Wahl L https://orcid.org/0000-0003-1163-0940

Wahlberg N

Wainwright P

Wakano J

Walasek N https://orcid.org/0000-0003-4411-319X

Waldock C

Waldrop L https://orcid.org/0000-0001-5708-2789

Walker A

Wallach A

Wall-Scheffler C

Walsman J https://orcid.org/0000-0001-8141-0340

Walter A https://orcid.org/0000-0003-0157-6151

Walters E https://orcid.org/0000-0002-9414-5758

Wang B

Wang S

Wang X

Wang Z https://orcid.org/0000-0002-8182-2852

Wang Z

Ward B

Wardell G

Wardle S https://orcid.org/0000-0003-2216-7461

Wares J http://orcid.org/0000-0003-1827-0669

Warlick A

Warton DI

Wascher C https://orcid.org/0000-0003-4360-363X

Wasser S

Watanabe J https://orcid.org/0000-0002-9810-5286

Waters M https://orcid.org/0000-0003-2660-3355

Watkins C https://orcid.org/0000-0002-1207-6331

Watson A https://orcid.org/0000-0002-9654-8147

Watve M https://orcid.org/0000-0003-0730-8393

Wcislo W

Webb J

Weber A https://orcid.org/0000-0002-7980-388X

Wedekind C https://orcid.org/0000-0001-6143-4716

Wee J

Wege M

Weiss A

Weissman J

Weithoff G

Welch KC

Wells K https://orcid.org/0000-0003-0377-2463

Werth A https://orcid.org/0000-0002-7777-478X

West K

Westley P

Westneat D

Westoby M https://orcid.org/0000-0001-7690-4530

Wheat CH

Wheat C

Whelan F https://orcid.org/0000-0001-9165-4859

While G

White H

White M

White T https://orcid.org/0000-0002-3976-1734

White W

Whitlock C

Whittington E

Wiberg A https://orcid.org/0000-0002-8074-8670

Wienecke B

Wiens J https://orcid.org/0000-0003-4243-1127

Wigby S https://orcid.org/0000-0002-2260-2948

Wiklund C

Wikman P

Wilber M

Wild G https://orcid.org/0000-0001-7821-7304

Wild S https://orcid.org/0000-0002-5904-0096

Wilhelm O

Wilkins A

Wilkins J https://orcid.org/0000-0001-6405-6538

Will W

Willi Y https://orcid.org/0000-0001-7654-3261

Williams C

Williams H

Williams T

Williams T https://orcid.org/0000-0002-6416-9441

Willian R https://orcid.org/0000-0003-2240-3970

Willing B

Wilson H

Wilson L

Wilson M https://orcid.org/0000-0003-3073-4518

Wilson R https://orcid.org/0000-0002-2567-3151

Wilson V https://orcid.org/0000-0001-7253-6013

Wilts B https://orcid.org/0000-0002-2727-7128

Winans J https://orcid.org/0000-0002-5658-0011

Winger B

Wissam J

Witkowski E

Witman J

Witt C https://orcid.org/0000-0003-2781-1543

Wittemyer G https://orcid.org/0000-0003-1640-5355

Wittman T https://orcid.org/0000-0002-8679-4782

Wojciechowski MS

Wolf A

Wolfner M

Wolinska J https://orcid.org/0000-0003-2913-2923

Wollrab S https://orcid.org/0000-0003-2430-4845

Wood L

Wood Z https://orcid.org/0000-0001-7369-9199

Woodbridge J

Woodcock B https://orcid.org/0000-0003-0300-9951

Worm B

Wright N

Wright R

Wu W

Wu Y https://orcid.org/0000-0002-6364-4597

Wurm Y https://orcid.org/0000-0002-3140-2809

Wystrach A https://orcid.org/0000-0002-3273-7483

Xing Y

Xu C https://orcid.org/0000-0002-1841-9032

Yamamichi M https://orcid.org/0000-0003-2136-3399

Yamashita N

Yampolsky L https://orcid.org/0000-0001-7680-6025

Yang Y https://orcid.org/0000-0003-2745-9498

Yang Z

Yao J

Yasuhara M https://orcid.org/0000-0003-0990-1764

Yeakel J https://orcid.org/0000-0002-6597-3511

Yijuan X https://orcid.org/0000-0003-1304-6289

Yoder J https://orcid.org/0000-0002-5630-0921

Yoeli E

Yonas A

Yorzinski J https://orcid.org/0000-0002-4193-6695

Yoshikawa T https://orcid.org/0000-0001-6998-275X

Yoshizawa M

Young M

Young R

Yovel Y

Yu HH

Yu L

Yuen K-V

Yukilevich R

Yusuf A https://orcid.org/0000-0002-8625-6490

Zahawi RA

Zamora S https://orcid.org/0000-0002-3917-4628

Zardoya R https://orcid.org/0000-0001-6212-9502

Zarzyczny KM

Zattara E https://orcid.org/0000-0002-9947-9036

Zavorka L https://orcid.org/0000-0002-0489-3681

Zeng L-Q

Zeng Q

Zettlemoyer M https://orcid.org/0000-0002-8203-7207

Zhang D-Y https://orcid.org/0000-0003-1056-8735

Zhang J https://orcid.org/0000-0003-0589-6267

Zhang L https://orcid.org/0000-0003-2409-7348

Zhang R

Zhang V

Zhang Z https://orcid.org/0000-0003-2090-7999

Zhao L

Zhe Q

Ziheng Y

Zilio G

Zimmer C

Zimmermann N

Zlatev J

Zoefel B

Zöttl M https://orcid.org/0000-0002-5582-2306

Zuberbühler K

Zuk M

Zuo L

